# SuperPhy: predictive genomics for the bacterial pathogen *Escherichia coli*

**DOI:** 10.1186/s12866-016-0680-0

**Published:** 2016-04-12

**Authors:** Matthew D. Whiteside, Chad R. Laing, Akiff Manji, Peter Kruczkiewicz, Eduardo N. Taboada, Victor P. J. Gannon

**Affiliations:** National Microbiology Laboratory @ Lethbridge, Public Health Agency of Canada, Lethbridge, AB T1J 3Z4 Canada

**Keywords:** Comparative genomics, Bioinformatics, Anti-microbial resistance, Virulence factors, Epidemiology, Population genomics, Software

## Abstract

**Background:**

Predictive genomics is the translation of raw genome sequence data into a phenotypic assessment of the organism. For bacterial pathogens, these phenotypes can range from environmental survivability, to the severity of human disease. Significant progress has been made in the development of generic tools for genomic analyses that are broadly applicable to all microorganisms; however, a fundamental missing component is the ability to analyze genomic data in the context of organism-specific phenotypic knowledge, which has been accumulated from decades of research and can provide a meaningful interpretation of genome sequence data.

**Results:**

In this study, we present SuperPhy, an online predictive genomics platform (http://lfz.corefacility.ca/superphy/) for *Escherichia coli*. The platform integrates the analytical tools and genome sequence data for all publicly available *E. coli* genomes and facilitates the upload of new genome sequences from users under public or private settings. SuperPhy provides real-time analyses of thousands of genome sequences with results that are understandable and useful to a wide community, including those in the fields of clinical medicine, epidemiology, ecology, and evolution. SuperPhy includes identification of: 1) virulence and antimicrobial resistance determinants 2) statistical associations between genotypes, biomarkers, geospatial distribution, host, source, and phylogenetic clade; 3) the identification of biomarkers for groups of genomes on the based presence/absence of specific genomic regions and single-nucleotide polymorphisms and 4) *in silico* Shiga-toxin subtype.

**Conclusions:**

SuperPhy is a predictive genomics platform that attempts to provide an essential link between the vast amounts of genome information currently being generated and phenotypic knowledge in an organism-specific context.

## Background

Whole-genome sequencing (WGS) of bacterial isolates generates the complete DNA sequence of each organism. WGS provides the greatest possible resolution of any typing method, the sequence is easily transferable, and its analyses can reveal important phenotypic insights such as the presence of virulence factors or anti-microbial resistance determinants. Current benchtop sequencers such as the Illumina MiSeq and the newly developed USB-sized Oxford Nanopore sequencer have made it possible for real-time WGS to be performed in the laboratory as well as on the front-line, as was recently seen in the 2014 Ebola outbreak, and in managing a hospital outbreak of *Salmonella* [1–4].

WGS will likely replace current typing and sub-typing methods due to its low cost, high information content, portability, and speed of analyses. It is now being used in real-time for: the identification of the source of foodborne outbreaks [5], surveillance [6, 7], epidemiological investigations [7], industrial applications [8, 9], population studies [10, 11], routine typing [12], regulation [13], providing point-of-care insight for clinicians [14, 15], informing veterinary practice [16], and helping inform public-health decisions [17].

WGS is now the *de facto* standard for bacterial strain analyses and the global community is coming together to help store and best utilize this rapid influx of information under the Global Microbial Identifier network (http://www.globalmicrobialidentifier.org/). This international effort currently involves 32 countries, many of which have their own national or regional programs to best utilize WGS data in public health, epidemiological and research contexts, such as the GenomeTrakR initiative of the Food and Drug Administration in the United States of America (http://www.fda.gov/Food/FoodScienceResearch/WholeGenomeSequencingProgramWGS/), the Integrated Rapid Infectious Disease Analysis (IRIDA) platform in Canada (http://www.irida.ca/), and the Patho-NGen-Trace project within the European Union (http://patho-ngen-trace.eu/project/).

Recently, several platforms have emerged that attempt to provide additional context in addition to the raw WGS data. For instance PATRIC provides pre-computed analyses for public genomes, including annotation, protein families, antibiotic resistance identification and comparative pathway analysis [18]. MicroScope provides an expert-guided annotation pipeline, as well as comparative analyses based on shared gene content [19]. The Integrated Microbial Genomes (IMG) project is also a combined genome annotation and analysis platform, that additionally allows for genomic data submissions by the user [20]. BIGSdb allows local comparisons among genomes using a multi-locus sequence typing approach, and allows phenotypic data to be stored along with the genomic information [21]. The Harvest suite of tools allows for fast core-genome alignments and interactive visualizations for thousands of genomes [22]. Other platforms focus on a specific organism, such as Sybil, a platform for the comparative analyses of *Streptococcus pneumoniae* based on BLASTP searches [23].

The large initiatives that generate and collect the tens- and hundreds-of thousands of genome sequences, and the platforms that host and analyze the public data provide an enormous benefit. Even though WGS and basic comparative analyses is commonplace, meaningful interpretation of the raw data in a phenotypic context, also known as predictive genomics, lags considerably behind [24]. Microbiologists often have organism-specific knowledge that can meaningfully inform the WGS data, but which is not incorporated into a generic analysis. The ability to interactively explore species-specific data that contains organism-specific knowledge from experts in the field is of tremendous value. A recent study on outbreak investigations using WGS also listed a main obstacle of routine adoption as ‘a paucity of user-friendly and clinically focused bioinformatics platforms’ [25]. While some components necessary for phenotypic prediction based on WGS data have been developed, there is currently no single integrated platform built to provide predictive genomic analyses for organism-specific end-users.

Here we present SuperPhy, a predictive genomics platform that brings organism-specific knowledge to comparative genomic analyses. SuperPhy incorporates knowledge from research on the pathogenesis and epidemiology of *E. coli*, as well as the tremendous amount of genotypic and phenotypic data that have previously been generated. This knowledge is used within SuperPhy to discover relationships among and about sub-groups. It allows non-bioinformaticians to quickly analyze new data against the background of other sequenced *E. coli*, facilitating novel insights.

We have previously developed Panseq, software that performs comparative genomics in a pan-genome context, identifying differences in the accessory genome and single nucleotide variations within the core genome [26]. SuperPhy utilizes the pan-genomic output from Panseq to identify: 1) virulence and antimicrobial resistance determinants 2) epidemiological associations between specific genotypes, biomarkers, geospatial distribution, host, source, and other metadata in an interactive and explorable setting; 3) statistically significant clade-specific genome markers (presence/absence of specific genomic regions, and single-nucleotide polymorphisms) for bacterial populations; and 4) *in silico* Shiga-toxin subtyping for genomes that possess *stx* genes.

SuperPhy allows the submission of genomes in a private or public context and is continually updated with the influx of public *E. coli* data from GenBank, allowing researchers to quickly analyze and compare new genomes with other publicly available sequenced *E. coli* strains. Predictive genomics provides an essential link between the vast numbers of genomes currently being generated and organism-specific phenotypic knowledge.

## Platform features

### Navigation and overview

The layout of the SuperPhy website (https://lfz.corefacility.ca/superphy) provides universal and quick access to the major components of the platform: ‘Group Analyses’ provides an interactive environment for comparing groups of strains based on metadata types or user-created strain-groupings, and determining statistically significant biomarkers for these groups (both the presence/absence of genomic regions and SNPs); ‘VF and AMR’ provides an ontology of both virulence genes and AMR determinants, and the ability to select groups of genomes and factors based on the provided ontologies. Output includes a summary of the presence/absence of selected VF and AMR factors among the strains of interest; ‘Group Browse’ provides an interface to examine groups of strains, and their distribution in both a geospatial and phylogenetic context simultaneously; ‘My Data’ provides an interface for uploading and modifying user-submitted genomes that are available only to the user; ‘Home’ provides a landing page and an overview of the features of the site. Additionally, an in-depth examination and report on an individual strain, including all known metadata, Shiga-toxin subtype (if applicable), phylogenetic and geospatial information, and a summary of virulence factor and anti-microbial resistance determinants can be accessed by selecting ‘detailed information’ from any genome in the platform.

### Strain selection

SuperPhy provides three methods of selecting *E. coli* genomes for analyses that are consistent across the site: list-, tree-, and map-based selections. The platform is based heavily on metadata, and as such provides a unified metadata control panel that displays the metadata fields and their associated values for each genome across each of the three views. The metadata control panel also allows filtering and selecting genomes that match given metadata criteria.

1. List-based selection provides a table-based interface to the genomes and their metadata, with private and public genome sets afforded their own sections.
2. Tree-based selection provides an interactive phylogeny that can be manipulated to expand/contract clades, and from which clade and individual genome selection can be made. Metadata is appended to each leaf node of the tree, and branches containing more than one genome have the metadata for the entire branch summarized as an interactive bar-chart that displays the frequency of values within selected metadata categories. This summary is an excellent way to visually discern clade differences, and allows an effective representation of thousands of genomes in tree form that would otherwise be intractable. An example of the phylogenetic tree with metadata clusters is shown in Fig. 1.

**Fig. 1:**
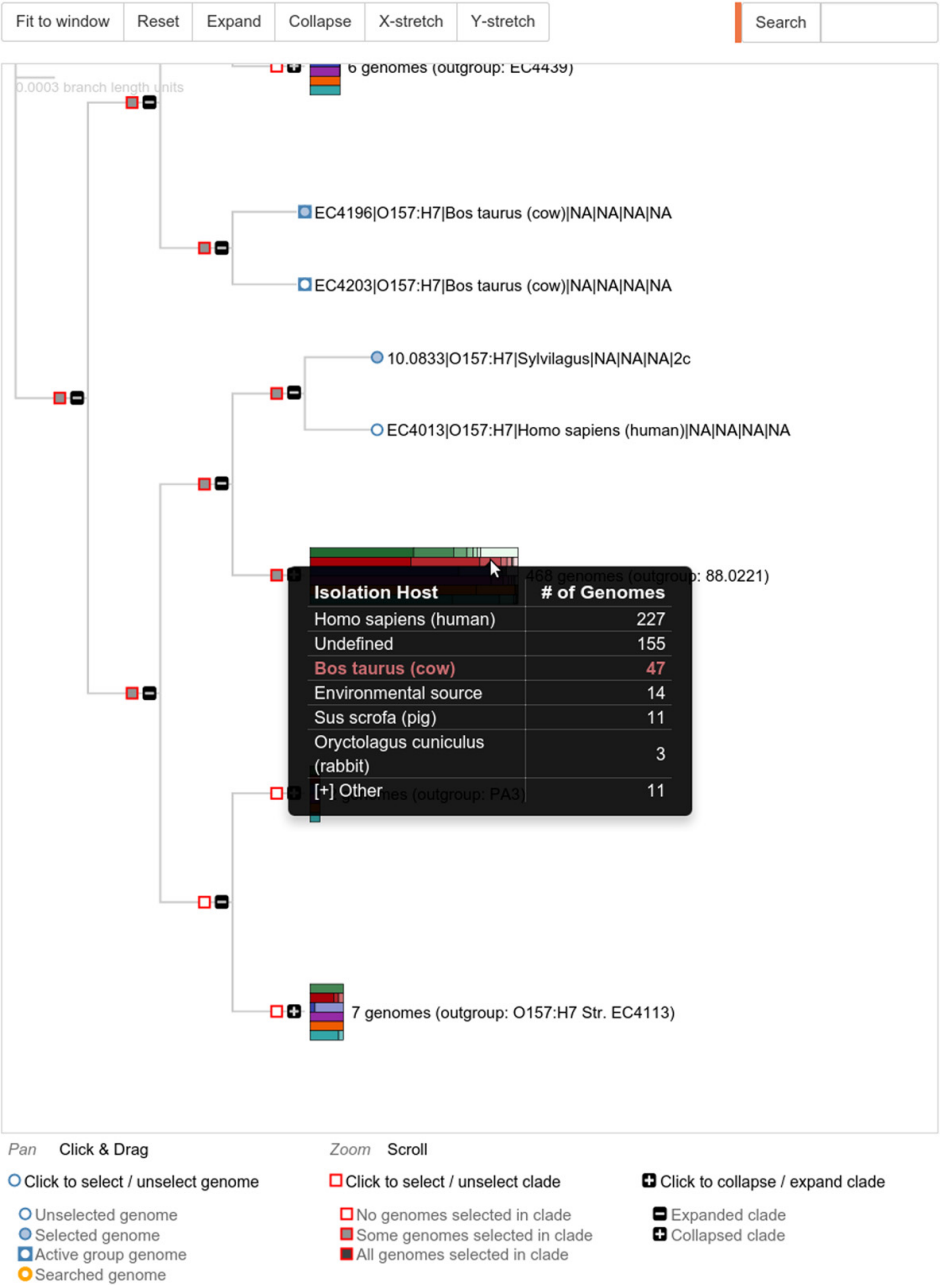
Interactive phylogeny with metadata. A screen capture showing tree-based selection from an interactive phylogeny that can be manipulated to expand/contract clades, and from which clade and individual genome selections can be made. Metadata is shown appended to each leaf node of the tree, and branches containing more than one genome have the metadata for the entire branch summarized as an interactive bar-chart. Each colored bar represents a metadata category, which is summarized in table form when highlighted; here the red bar representing Isolation Host is shown with a frequency table of hosts. Metadata represented as bars are as follows: Green:Serotype, Red:Isolation Host, Blue:Isolation Source, Purple:Symptoms/Disease, Orange:Stx1-subtype, Teal:Stx2-subtype
3. Map-based selection provides a Google Maps interface to geospatial genome selection, along with a table-view of the metadata for the genomes in the map. Just as in the list-based view, the displayed metadata fields for each genome can be changed, and used to filter the displayed genomes. As an example, we show the map when a user searches for ‘United Kingdom’ in Fig. 2.

**Fig. 2:**
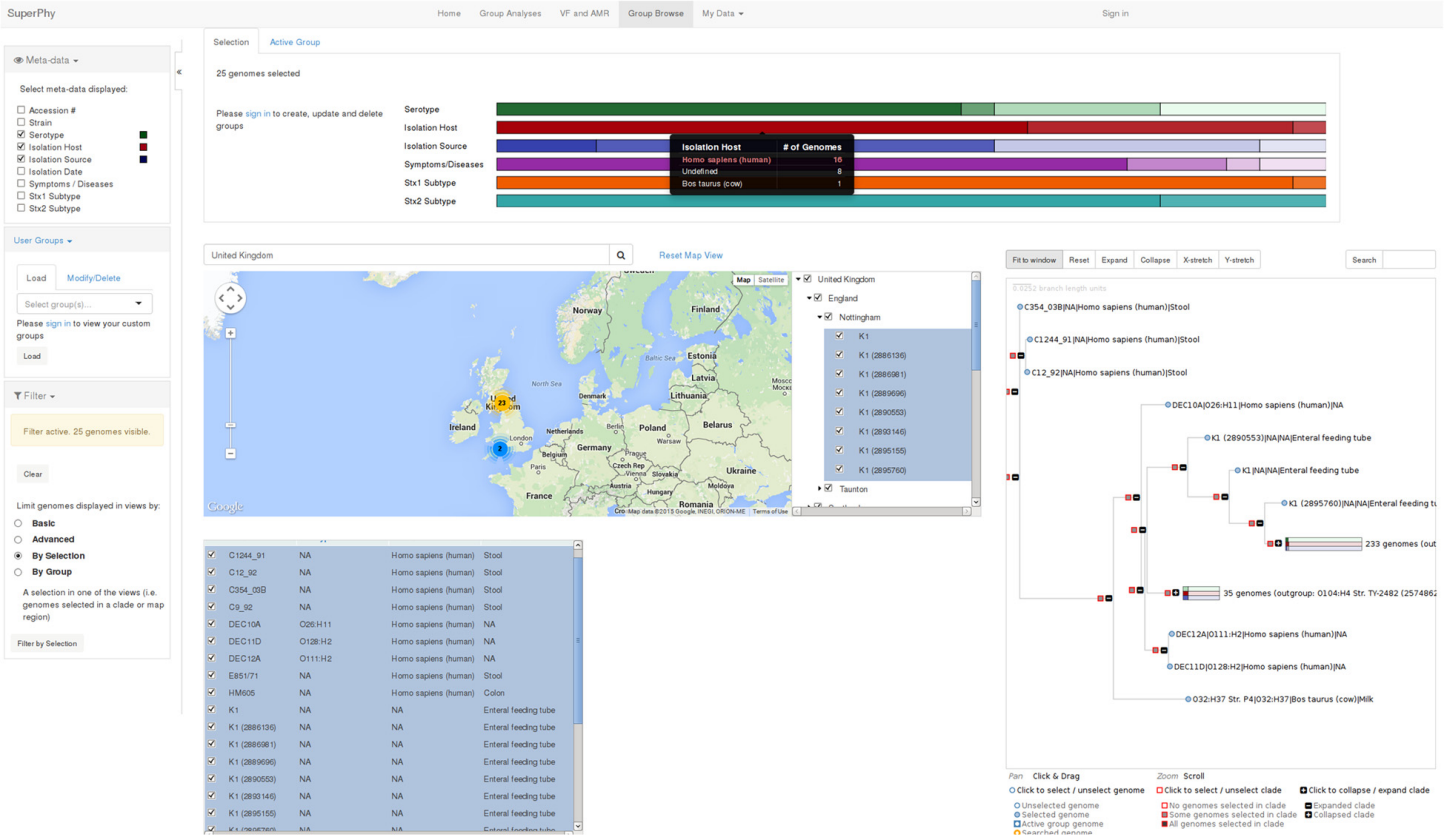
Map-based group selection of United Kingdom genomes. A screen capture showing selection of a group of genomes based on the map interface. In this example, the search term ‘United Kingdom’ has been used to focus the map on the respective world region, which displays a hierarchical view of regions and subregions visible in the map. Here, the ‘United Kingdom’ checkbox has been used to select all subregions and genomes below it in the hierarchy eg. ‘Nottingham’ and the genomes from that region. The three views (tree, map, and list) have been filtered to display only the genomes from the ‘United Kingdom’, and the top of the page displays a metadata breakdown of the currently selected genomes for all metadata, where each colour represents a metadata category, shades of that colour represent separate values, and the size of the shaded bar represents the percentage of the total genomes with that value. The display is interactive, and hovering over a metadata category presents a summary table, as shown here for ‘Isolation Host’

### Website usage tutorials

The main pages of the SuperPhy platform include a guided tutorial introduction using the IntroJS plugin (https://usablica.github.io/intro.js/). This tutorial provides a walk-through of all the major features and how to use them, and is activated by clicking the large red ‘Introduction’ button located on each page.

## Implementation

### Webserver application and database

Genome data and analyses are administered using a PostgreSQL 9.3 database with a schema adapted from the Generic Model Organism Database (GMOD) Chado schema [27]. The Chado relational database schema uses a flexible, ontology-centric approach to organizing biological entities, relationships, properties and analyses. Entries in generic tables are assigned types using a mutable, controlled vocabulary. By not defining entity types directly into the relational layer, the database can be highly adaptable and can grow to add new analyses or biological data.

The application layer for the SuperPhy website is build using the Model-View-Controller (MVC) Perl CGI::Application framework (http://www.cgi-app.org/). The phylogenetic tree display and interaction is built on top of the Data Driven Documents (D3) JavaScript library (http://d3js.org/). Geospatial views are built using the Google Maps JavaScript API v3 (https://developers.google.com/maps/documentation/javascript/). Group comparisons are processed and displayed using the RStudio Shiny web application framework for R [28].

The webserver application code base, database schema and public data are hosted on Github at https://github.com/superphy/version-1.

### Access to uploaded data

Users can upload genomes and metadata and choose between three access levels to govern their use: ‘public’ information is available to all users; ‘private’ information is only available for the genome uploader and additional users they select; and ‘private until a specified date’ data is released to ‘public’ data after a specified date. Users may also designate other registered users for whom the data will be available. Private data is accessible only to designated users, but can be combined with public data for user-specific analyses. Users can create custom genome-groups that can be saved, and all results may be downloaded for offline analyses.

Uploaded data undergo a series of checks to ensure the quality of the data. Data are rejected if any of the following conditions are met: 1) Greater than 1000 contigs; 2) Genome size less than 3 Mbp or greater than 7.5 Mbp; 3) Invalid nucleotide characters (all IUPAC characters are valid); 4) The MD5 checksum of the concatenated contigs already exists in the database; 5) The SNP string for the pan-genome alignment is identical to another strain in the database.

Uploaded genomes undergo two checks to ensure the data are of a minimum quality, and that the genomes being uploaded contain markers that were found to be present only in genomes of *E. coli*. We initially identified genomic regions present in at least 70 % of the genomes, referred to as the “conserved core”. All genomes are considered to be *E. coli* if: 1) they contain at least 1500 conserved core regions, and 2) The presence of at least three *E. coli* species-specific regions. The derivation of these markers is presented in the ‘Pan-genome’ subsection of the ‘Example analyses’.

### Acquisition of public *Escherichia coli* genomes

SuperPhy is continually and automatically updated with closed and draft genomes of *Escherichia coli* from GenBank using the script https://github.com/superphy/version-1/Sequences/ncbi_downloader.pl. All metadata present in the GenBank submissions are extracted automatically using the script https://github.com/superphy/version-1/Sequences/genbank_to_genodo.pl. For the initial bulk upload, a second phase of manual curation was carried out to ensure all available metadata was included, even if it was stored in a non-standard way during the initial submission. The complete list of 1641 public *E. coli* genomes present in the SuperPhy database at the time of manuscript preparation, along with all extracted metadata is available at (https://github.com/superphy/version-1/Data/metadata_table.csv). A summary of the metadata fields used in SuperPhy, as well as the percentage of the public genomes containing information for a particular metadata category is presented in Table 1.

**Table 1:** The percentage of genomes that contain metadata for each of the metadata fields in the initial public data set of 1641 *E. coli* in the SuperPhy database

Metadata field	Percentage
Location	85
Host	79
Date of Isolation	63
Source	52
Serotype	44
Stx2 subtype	23
Stx1 subtype	18
Disease syndrome	6

### Comparative genomic analyses

Our pan-genomic analyses tool, Panseq is used for the background comparative analyses [26]. It iteratively adds new genomic sequences, and compares them to those already stored in the platform. This computational approach allows a continuous influx of new sequence data without large time or memory requirements. In this way, the complete pan-genome of all sequences in the database is determined. Annotations for these regions are determined by querying the GenBank NR protein database via BLASTx.

Differences in the accessory genome and the single nucleotide variation in the core genome are obtained and used by SuperPhy in downstream applications including the construction of discriminatory and robust phylogenies, and in the pre-computed data for bio-marker identification among groups of genomes.

### Tree construction

SuperPhy provides a global phylogenetic tree that is updated to include all *E. coli* genomes currently in the database. An initial phylogenetic tree for SuperPhy was constructed using conserved genomic regions from the 1641 *E. coli* genomes obtained from GenBank. The conserved regions were aligned using Muscle [29] and input into FastTreeMP to build a minimum-evolution tree [30]. To achieve sufficient resolution in branch lengths to disambiguate strains, the double-precision version of FastTree was used [30]. As new genomes are uploaded to SuperPhy, they are incorporated into the multiple sequence alignment and a new tree is rebuilt, which becomes the tree used for all analyses within the SuperPhy platform.

### Virulence and anti-microbial resistance markers

The presence/absence of virulence and AMR genes are computed using Panseq. The non-redundant query set of AMR genes from the Comprehensive Antibiotic Resistance Database (CARD) [31] is used for *in silico* AMR determinant screening. All AMR genes are organized and stored in the database according to their CARD-assigned Antibiotic Resistance Ontology annotation to aid in identifying the presence of different antimicrobial resistance mechanisms. The virulence gene database was constructed by obtaining all gene alleles of known virulence factors for *E. coli* from the Virulence Factor Database [32], supplemented with additional virulence factors from ‘*Escherichia coli*: Pathotypes and Principles of Pathogenesis, 2nd Ed.’ [33], and additional published literature, which effectively doubled the number of virulence factors in the database. To avoid duplication of factors, all AMR and virulence factor sequences were clustered based on similarity using BLASTclust with default settings; the longest allele was selected for each gene, except in cases where sequence similarity was less than 90 %, in which case multiple alleles were included [34].

**Fig. 3:**
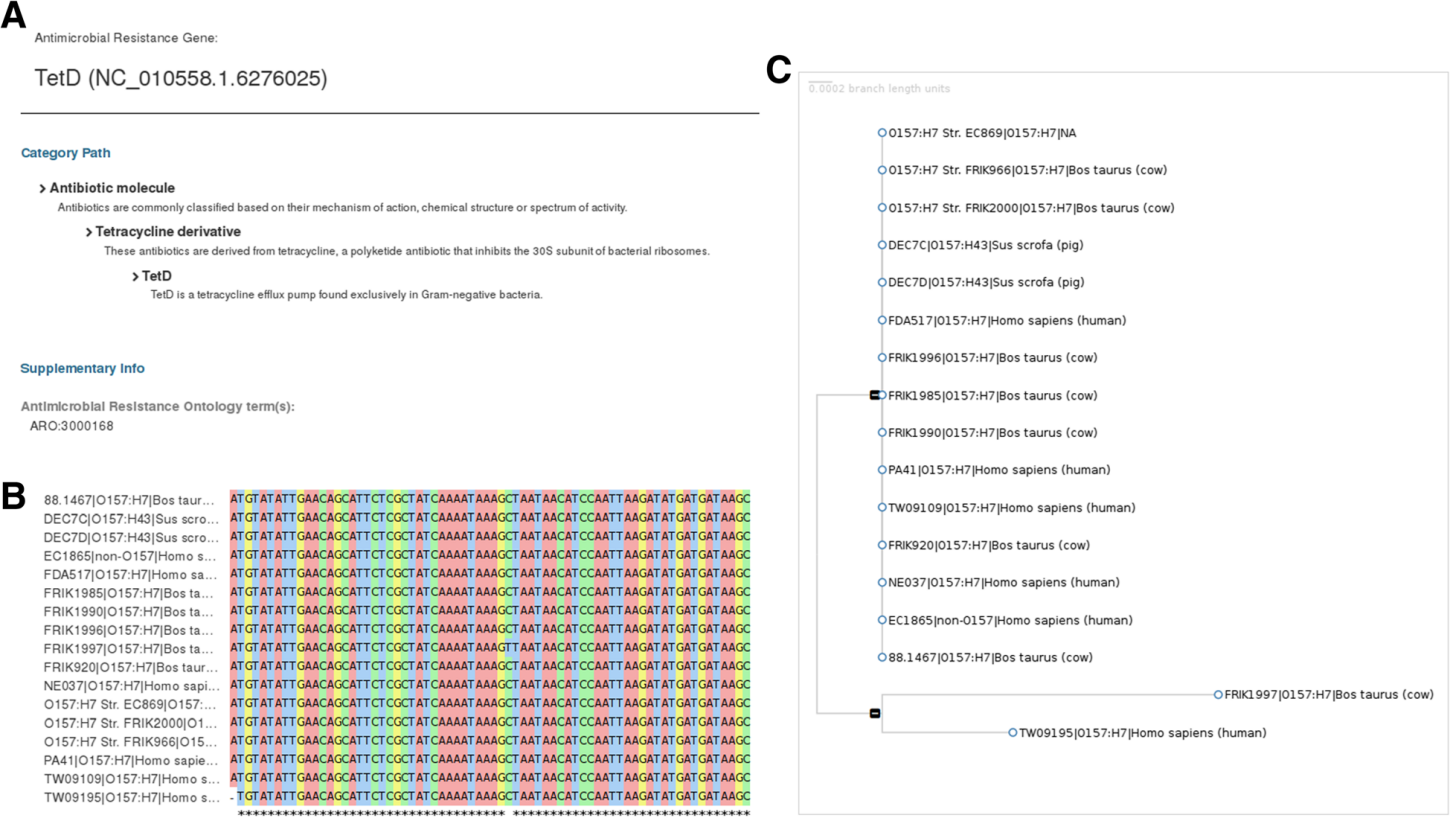
Phylogeny and multiple-sequence alignment of the gene *tetD* within the SuperPhy platform. Combined screen captures showing contextual information (**a**), multiple sequence alignment (**b**) and accompanying phylogenetic tree (**c**) for the gene *tetD*, for a subset of serogroup O157 genomes in the SuperPhy database that contain a copy of the gene. Both the tree and the sequence alignment are interactive

In addition to providing the presence/absence of virulence and AMR factors, SuperPhy stores the sequence of the individual alleles for each genome, and constructs a phylogeny based on each single gene. This allows one to compare the relationships among genomes based on a single virulence or AMR attribute and to examine the sequence variation of the gene at the individual base level, as the multiple sequence alignment (MSA) can also be displayed, as shown in Fig. 3.

### Group comparisons

The statistical identification of markers that differ between groups based on both single nucleotide polymorphisms and the presence/absence of genomic loci is implemented using a two stage approach: 1) To rapidly assess the thousands of possibilities, the ‘approximate’ vectorized Fisher’s Exact Test (FET) from the R corpora package is calculated (http://cran.r-project.org/web/packages/corpora/index.html), following which the exact FET value is computed for the 100 most-significant results using the FET function from the base R statistical package [35]. The comparisons are corrected for multiple-testing using the false-discovery rate method of Benjamini and Hochberg. All single-nucleotide polymorphisms and genomic presence/absence data reside in the database, and require only the retrieval and *P*-value computation for the strains of interest for the real time analysis of genome markers.

The R Shiny interface is used for group creation and all metadata fields are pre-populated for all strains in the database. This makes comparing, for example, all human and non-human strains of a given serotype as simple as selecting groups based on the serotype and host metadata fields, and clicking the compare button. Additionally, custom groups of any genomes can be created and saved to a user-profile so they become available whenever the user is logged in. These custom groups can include private genomes available only to the logged-in user, in addition to any public genomes.

### Stx typing

Shiga-toxin (Stx) subtype assignment, when a strain possesses a copy of one or more of *stx1* or *stx2*, is calculated based on a phylogenetic tree generated from concatenated and aligned a and b subunits for each of Stx1 and Stx2. Clades specific to a Shiga-toxin subtype were identified based on the scheme presented by Scheutz et al. (2012) [36]. Membership in these pre-defined clades is used to identify the subtype of the toxin gene; those strains that fall outside of known sub-type clades are marked as unknown. Multiple sequence alignments of the Stx genes are stored in the database for reference and comparison.

### Geospatial visualization

The geospatial visualizations provide an interactive map interface for selecting and and searching genomes and groups of genomes. SuperPhy leverages Google Maps along with the companion Javascript library, Google Maps API (V3).

Genome location data is geocoded for latitude and longitude during the process of adding a new strain to the platform. To reduce the computational overhead in rendering thousands of genome map markers, the marker clustering algorithm MarkerClusterPlus for Google Maps V3 (http://google-maps-utility-library-v3.googlecode.com/svn/trunk/markerclustererplus/docs/reference.html) was implemented. Locations within a distance of 60 pixels on the map are clustered into a single marker rendered at the geometric center of the cluster, and a count of the number of genomes is displayed.

All geospatial views are accompanied by a dynamic and sortable table of genome metadata that is by default sorted by country. Users also have the option of sorting by province, state and city. The table is dynamic and updates to display information for the genomes visible on the map. Locations for each *E. coli* strain can be downloaded for offline manipulation.

### Continuous integration

The user community is able to provide feedback as the platform evolves in the form of feature requests and bug reports using the ‘Issues’ section at https://github.com/superphy/version-1/issues. This will ensure the platform evolves in a way that is most beneficial to those who use it.

## Results and discussion

### Pan-genome

At the time of writing, 2324 publicly available *E. coli* genomes from GenBank had been analyzed for incorporation into the SuperPhy platform [37]. *E. coli* is a ubiquitous, Gram-negative bacterial species found in the intestines of healthy mammals, with only a small subset causing disease in humans or animals [38]. The population structure of *E. coli* was initially described as being broadly distributed among four large and two smaller phylogenetic groups [39, 40]. Previous studies have found that the species has an open pan-genome, meaning that the addition of new genomes is likely to add additional genes to the pool [41]. The pan-genome of *E. coli* is highly variable, with around 80 % of an individual genome comprised of accessory genes and the remainder from the shared core genome [42]; a stable proportion of approximately 4000 genes are present in at least 50 % of the genomes [43].

**Fig. 4:**
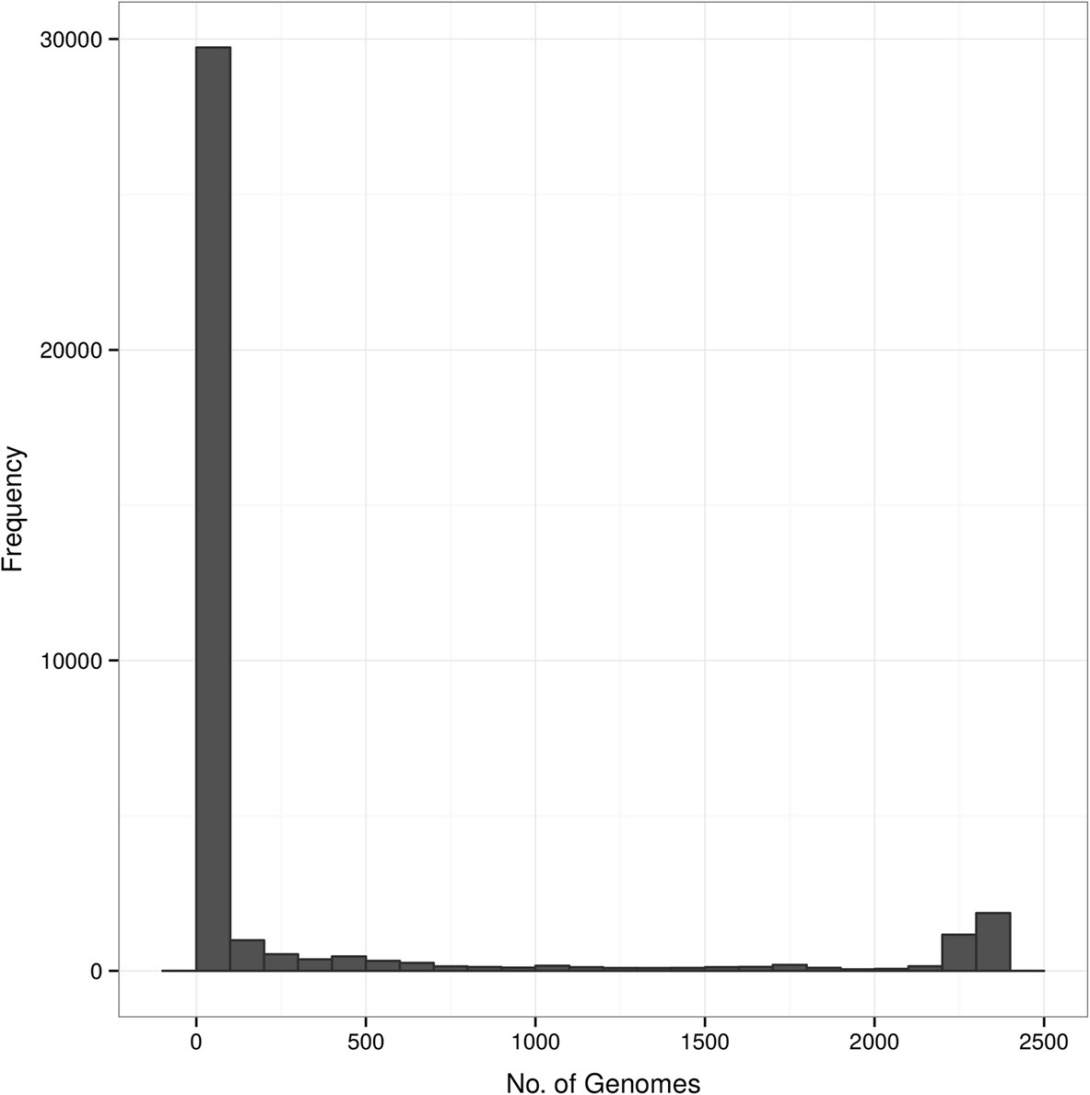
The pan-genome distribution among 2324 *E. coli* genomes. The pan-genome distribution of 2324 *E. coli* genomes as 1000 bp genomic segments. The majority (29.7Mbp) of the 37.44 Mbp pan-genome is present in fewer than 100 genomes, with the core genome size (present in at least 2300 genomes) observed to be 1.86Mbp. Only 5.84Mbp of the pan-genome was found in greater than 100 genomes, but fewer than 2300 genomes. Of these 2324 genomes, only 1641 had metadata beyond the name of the strain

The pan-genome distribution of these 2324 *E. coli* genomes as 1000 bp genomic segments is presented in Fig. 4. As can be seen, the majority (29.7 Mbp) of the 37.44 Mbp pan-genome is present in fewer than 100 genomes, with the core genome size (present in at least 2300 genomes) observed to be 1.86 Mbp. Only 5.84 Mbp of the pan-genome was found in greater than 100 genomes, but fewer than 2300 genomes. Based on these results, we selected a ‘conserved core’ of 3598 genomic regions, defined as those present in at least 70 % of the 2324 genomes. The conserved core is used within SuperPhy to identify SNPs that are used in phylogenetic tree building, as well as in the quality filtering of uploaded genomes.

Additionally, we endeavored to identify genomic regions that were specific to the species *E. coli*. To achieve this we screened the ‘conserved core’ against genomes from a subset of *E. coli* and other bacterial species, the results of which are presented in Table 2. The *E. coli* genomes contained more of the ‘conserved core’ regions than any of the other genomes examined, although genomes from *Shigella spp.* contained nearly as many, which is not surprising given that *Shigella spp.* has long been known to be very similar to *E. coli* [44]. Recent work using the analyses of whole genome sequence data of both *Shigella spp.* and *E. coli* showed *Shigella spp.* to form three separate monophyletic clades within the *E. coli* species [45], and that there was a mixing of traditional *Shigella spp.* within these clades. The analyses that we performed in the current study to find *E. coli* specific regions treated *Shigella spp.* as distinct from *E. coli*; had we considered them as sub-groups within *E. coli*, the number of species-specific markers would likely have increased.

**Table 2:** The number of conserved core genomic regions present in 19 selected bacterial genomes, from the total 3598 conserved core genomic regions found in at least 70 % of the 2324 *E. coli* genomes examined

Genome	No. ‘conserved core’ genes
*E. coli O103:H2,12009*	3563
*E. coli O157:H7, EDL933*	3557
*E. coli K-12, MG1655*	3550
*E. coli, UMN026*	3483
*E. coli O7:K1, CE10*	3448
*E. coli O83:H1, NRG 857C*	3289
*Shigella sonnei, 53G*	3259
*Shigella flexneri 2002017*	3148
*Shigella boydii, CDC 3083-94*	2965
*Shigella dysenteriae, 1617*	2683
*Escherichia fergusonii ATCC 35469*	1619
*Salmonella enterica subsp. Enterica serovar Typhimurium str. 14028S*	95
*Citrobacter rodentium, ICC168*	77
*Klebsiella oxytoca, E718*	50
*Klebsiella pneumoniae subsp. Pneumoniae, 1084*	50
*Klebsiella variicola, At-22*	46
*Escherichia blattae, DSM 4481*	27
*Staphylococcus aureus, 04-02981*	0
*Listeria monocytogenes, 07PF0776*	0

The results shown in Table 2 were filtered based on the distribution among these 19 genomes to identify genomic regions present in only the *E. coli* genomes, resulting in 33 candidates; the raw data table is available at https://github.com/superphy/version-1/Sequences/genome_content_panseq/binary_table.txt. These 33 candidates were screened against the GenBank ‘nr’ and ‘WGS’ databases using the ‘bacteria’ taxid to limit the search; the raw BLAST results are available at https://github.com/superphy/version-1/Sequences/genome_content_panseq/UB0HWGTR015-Alignment.xml and https://github.com/superphy/version-1/Sequences/genome_content_panseq/UD4GVA26015-Alignment.xml. Based on these queries using a 90 % total sequence identity threshold, we removed all putative species-specific regions that were identified in genomes from bacteria other than *E. coli*, and were left with the ten species-specific regions presented in Table 3.

**Table 3:** The ten *E. coli* species-specific genomic regions identified in this study based on a total sequence identity of 90 %, their location in the K12 reference genome MG1655, the number out of 2324 *E. coli* genomes each region was found in, and their putative function based on the top scoring BLASTx hit

Region ID	Start bp	End bp	No. genomes	Putative function
*3160548*	347258	346259	2238	Propionate catabolism operon regulatory protein PrpR
*3160296*	537566	536567	2256	2-hydroxy-3-oxopropionate reductase
*3160113*	538566	537567	2248	Allantoin permease
*3159571*	541565	540567	2275	Purine permease ybbY
*3159389*	542566	541567	2268	Glycerate kinase
*3158844*	545665	544666	2261	Allantoate amidohydrolase
*3158667*	546665	545666	2272	Ureidoglycolate dehydrogenase
*3159808*	1588200	1587201	2171	FimH protein
*3160196*	4411062	4410063	2261	Hypothetical protein
*3158082*	4456632	4457631	2074	Mur ligase family, glutamate ligase domain protein

**Fig. 5:**
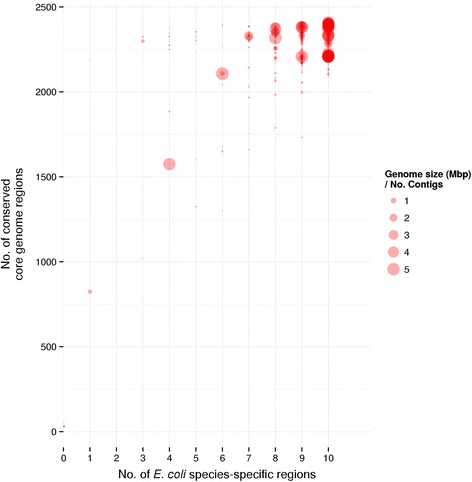
Correlation between species-specific regions and genome quality. The correlation between the presence of the ten species-specific regions and the 3598 ‘conserved core’ genomic regions identified in this study, among 2324 *E. coli* genomes. Genomes are plotted as dots where the size of the dot reflects genome quality, given by “genome size (Mbp)”/“No. contigs”

The correlation between the species-specific regions and the ‘conserved core’ regions among the 2324 *E. coli* genomes is presented in Fig. 5. As can be seen, not all species-specific markers were found in all strains; however, most *E. coli* genomes contained at least 8 of the markers and all contained at least 3 given the quality checks for assembled genomes previously described. A general trend was observed where genomes with higher ratios of ‘Genome size’/‘No. contigs’ contained both more ‘conserved core’ regions and species-specific regions, indicating that the quality of genome assembly affects the number of genomic regions that can be identified at a given sequence identity threshold. Based on these results, any genome in the SuperPhy database is defined as *E. coli* if it possesses at least three of the species specific markers and at least 1500 of the conserved core genomic regions.

Of the 2324 genomes examined, only 1641 had metadata beyond the name of the strain. As such, the initial SuperPhy database contained only these 1641 genomes to facilitate a metadata driven approach to genomic analysis.

### Predictive markers for sub-groups

A ‘group’ of bacteria can be defined in numerous ways, from spatially or temporally co-located strains, to those sharing biochemical utilization patterns, or those that occupy a clade of a phylogenetic tree. Regardless of how a group is defined, users are generally interested in defining characteristics that are predictive of the group, and can be used to discriminate its members from those of other related genomes. SuperPhy utilizes both the presence/absence of genomic regions, and SNPs within shared regions to define markers statistically predictive of a group. These identified biomarkers have potential downstream application in *in silico* diagnostics or simple wet-lab tests for the identified markers.

**Fig. 6:**
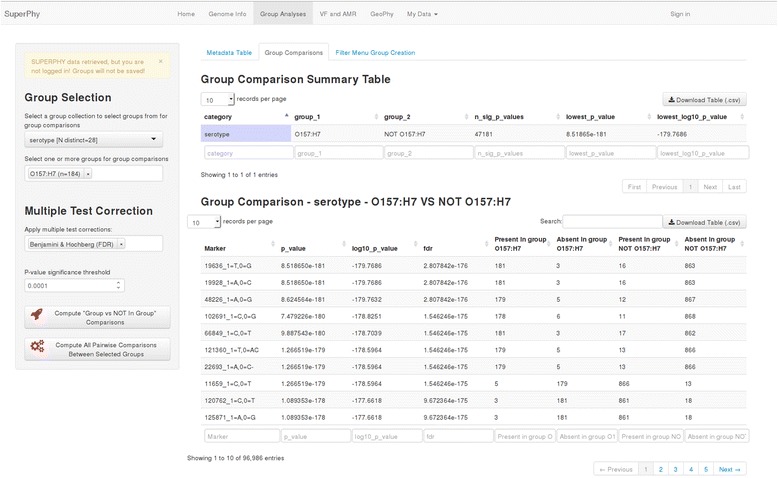
Group analyses identifying O157:H7 predictive SNPs. A screen capture demonstrating the ‘Group Analyses’ functionality of SuperPhy. In this example, all genomes of serotype O157:H7 are compared to all other genomes, and SNPs in the shared regions are ranked by *p*-value, from most statistically predictive of the group to least, with false discovery rate multiple testing correction. The results table is interactive and the complete dataset can be downloaded as a .csv file for offline analyses

As an example, we utilized the ‘Group Analyses’ feature of SuperPhy to identify SNPs that were statistically predictive for *E. coli* of serotype O157:H7 with respect to those of all other *E. coli*. This is demonstrated in Fig. 6, where the SNPs are ranked from most- to least-significant. The marker ID for each SNP, the polymorphism being examined, the *p*-value, the false discovery rate adjusted *p*-value, and the presence/absence of each SNP for the two groups being examined are displayed. The marker ID provides a link to a ‘SNP Information’ page, which identifies the pan-genome region the SNP is found in, the allele frequency of SNPs for all genomes in the database, the putative function of the region given by the top BLAST hit, and an option to download detailed SNP information for each genome. The download includes the genomic location, allele, and upstream/downstream sequences for all genomes in the database.

In addition to providing groups based on metadata categories such as serotype, and providing group vs. non-group comparisons, SuperPhy allows multi-way group vs. group comparisons. For example, if ‘isolation host’ is selected, then the categories ‘Bos taurus (cow)’, ‘Homo sapiens (human)’, and ‘Environmental source’ are used to generate comparisons between all combinations of the categories. This facilitates more rapid identification of group and sub-group predictive markers for the genomes being examined.

### Distribution of the *eae* gene

**Fig. 7:**
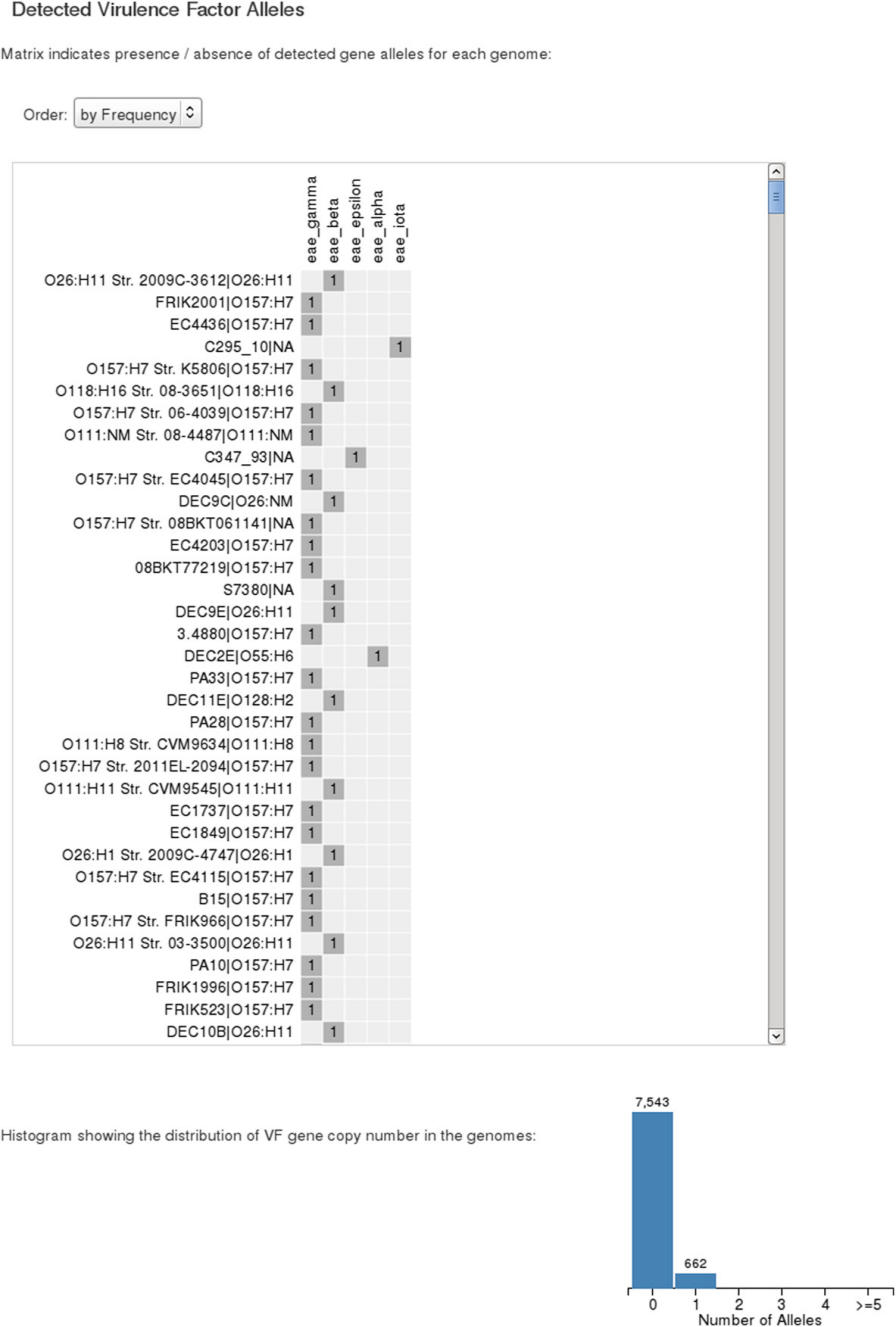
Virulence factor analyses in SuperPhy. A screen capture showing the matrix representation of all genomes that contain an allele of the *eae* gene. The matrix contains more data than can be displayed in a single image, but it is interactive and scrollable, allowing the full matrix to be explored by the user. The metadata category ‘Serotype’ has been activated and can be seen as appended to the strain name in the row names of the matrix. The numbers within the matrix indicate the copy number of an allele within a genome, and empty boxes indicate the absence of an allele. The histogram displays the copy number of all genes searched for; in this case, the number of *eae* alleles

Within the species *E. coli*, there are a subset of strains that attach to human intestinal epithelial cells via an attaching and effacing mechanism, the requisite apparatus for which is encoded in a genomic island known as the locus of enterocyte effacement (LEE) [46]. As an example of the ‘VF and AMR’ functionality within SuperPhy, we identified the distribution of the LEE gene *eae* among the 1641 public genomes in the SuperPhy database. All virulence factors are stored using controlled ontologies, which facilitate easy addition and retrieval of related data. The ontological category ‘LEE-encoded TTSS effector’ provided the *eae* alleles, and they were selected, along with all 1641 public genomes. The results are presented in an interactive matrix of gene presence/absence, as well as allele copy number (Fig. 7). Within the 1641 genomes examined, 662 possessed any of the 11 known variants of the *eae* gene at a sequence identity cutoff of 90 %. Additionally, SuperPhy provides a table of the results for download, where subsequent offline manipulation is possible.

### Analyses of geographical and phylogenetic clusters

**Fig. 8:**
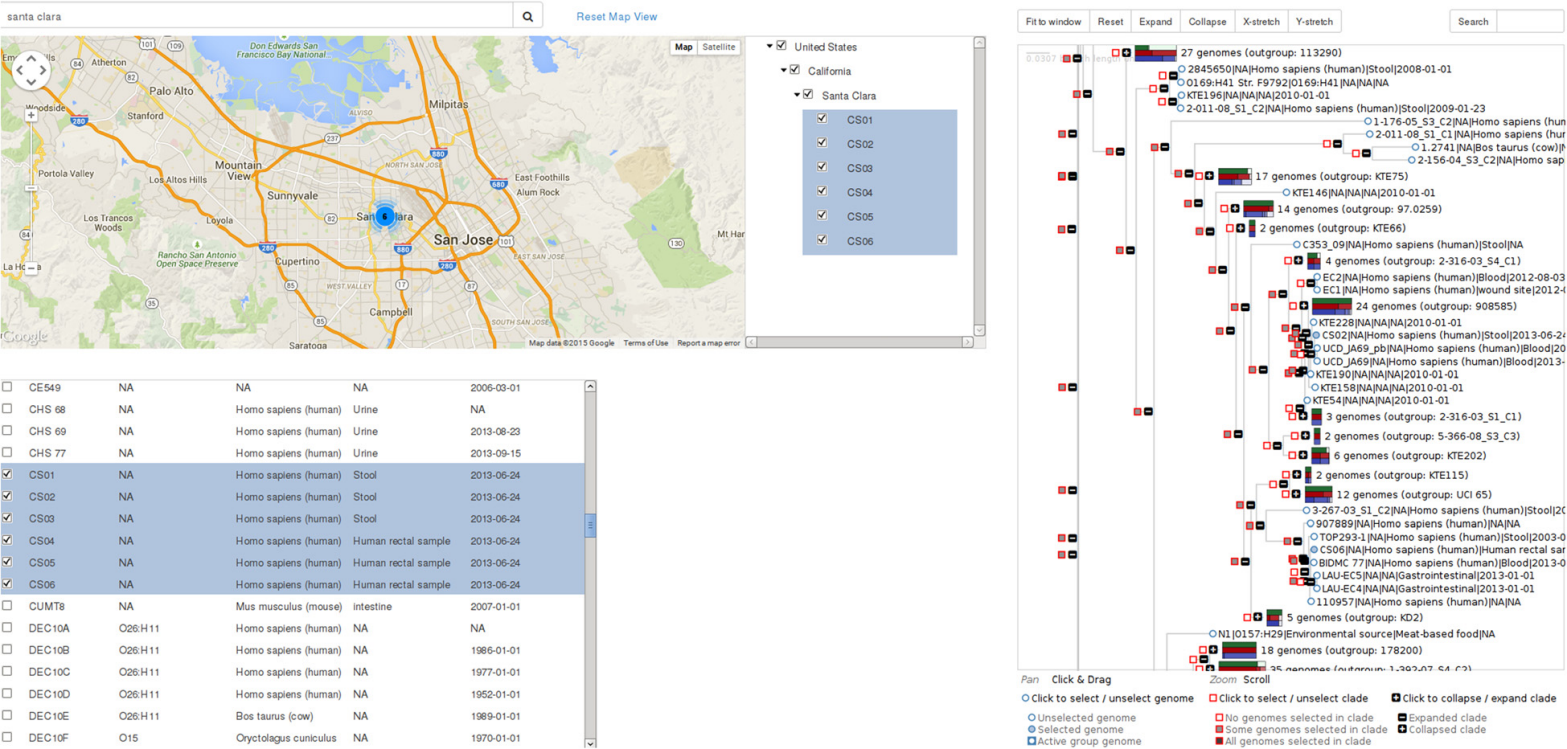
Simultaneous geospatial and phylogenetic analyses. A screen capture showing the ability to group genomes by geographical location and simultaneously examine their phylogenetic position. In this example, all six genomes from Santa Clara, California, United States are selected and highlighted in the map, tree and list views. The available metadata shows that all six genomes were isolated from human sources on the same day; however, their phylogenetic positioning indicates that they are not all from a clonal source. On the tree, all nodes that contain a selected genome are shown as blue-filled squares, while those that do not are white filled squares. Similarly, all selected genomes appear on the tree as blue-filled circles, and those not selected as white-filled circles

The ‘Group Browse’ section of SuperPhy provides a means for selecting, filtering and exploring groups of genomes utilizing the three modes of genome selection, namely the tree, map and list views. These allows users to view geographical clusters in terms of their corresponding position in a phylogenetic tree. For example, using the map view, and the hierarchical listing of locations, all genomes with the isolation location of Santa Clara, California, United States were selected and their corresponding positions on the phylogenetic tree automatically highlighted, as shown in Fig. 8 . Here it is evident that although all six genomes were isolated from Santa Clara, California on the same day, the genomes do not form their own cluster on the phylogenetic tree. On the tree, all nodes that contain a selected genome are shown as blue-filled squares, while those that do not are white-filled squares. Similarly, all selected genomes appear on the tree as blue-filled circles, and those not selected as white-filled circles. The six selected genomes from Santa Clara are widely distributed throughout the tree (at this zoom level, they are not all visible). Genomes CS02 and CS06 are both visible, on separate branches of the tree, indicating they are less related to each other, and the other four genomes from Santa Clara, than several other *E. coli* genomes with which they group more closely.

This ability to quickly examine geographical strain clusters in a phylogenetic context would prove extremely useful in determining if a group of genomes from the same time and place originated from a single bacterial clone, as in an outbreak situation or in the routine surveillance of a location such as a food-processing plant, to determine whether bacterial isolates were that of a persistent strain.

**Fig. 9:**
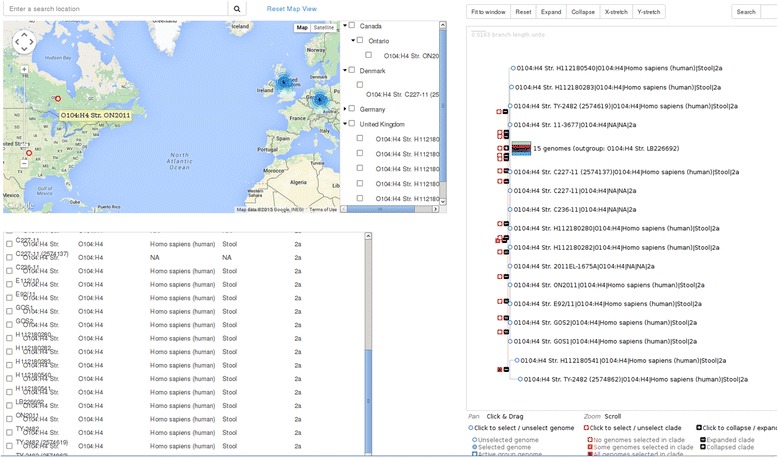
Global spread of 2011 O104:H4 outbreak strains. A screen capture showing genomes from the *E. coli* O104:H4 outbreak that occurred in Germany in 2011. The phylogeny of the outbreak strains shows their clonality, and the metadata, visible on the map, shows the dissemination of the bacterial clone from the German epicenter to countries such as Denmark, the United Kingdom, Canada, and the United States, which were determined to be travel-acquired infections

Conversely, within SuperPhy one can also select a phylogenetic clade and have the geographical locations of all strains shown. The ability to break apart a cluster of strains that are related at the genome level into geographical and metadata categories has use in source tracking of strains, and in determining the geographical dissemination of bacterial clones over time. As an example, genomes from the serotype O104:H4 outbreak that occurred in Germany in 2011 were chosen. This outbreak was the first caused by strains of O104:H4 that were found to have acquired the *stx2* gene through lateral gene transfer, which is thought to have been the contributing factor that led to the high rates of acute illness in healthy adults observed throughout the outbreak [47]. As can be seen in Fig. 9, the O104:H4 strains containing the *stx2* gene are nearly identical on the phylogenetic tree; however, the source of isolation of these bacteria, visible on the map, shows the dissemination of the bacterial clone from the German epicenter to countries such as Denmark, the United Kingdom, Canada, and the United states, which were determined to be travel-acquired infections.

## Conclusions

Predictive genomics and platforms that easily facilitate it are poised to become the translation layer between the vast amounts of sequence data and biological knowledge in a specific domain that is needed to test hypotheses. SuperPhy allows users to make some of these genotype/phenotype correlations, and platforms like it will become increasingly important in transforming raw genome data into useful knowledge.

Current work involves the addition of previously published *in silico* serotyping schemes to SuperPhy, and the expansion of the platform to include the bacterial pathogens *Salmonella enterica* and *Campylobacter jejuni*. Lastly, a representational state transfer (REST) application programming interface (API) is being designed to allow programmatic interaction with the SuperPhy platform, which will help ensure that SuperPhy does not become a data silo but can instead contribute to a dynamic and growing web of biological knowledge.

## Availability and requirements

**Project name:** SuperPhy

**Project home page:**https://lfz.corefacility.ca/superphy

**Operating system(s):** Platform independent (modern web-browser; the most recent Firefox or Chrome for best experience)

**Programming languages:** Perl, Coffeescript/Javascript, R

**License:** Apache2

## Availability of supporting data

The project is entirely open source under the Apache 2 license (https://www.apache.org/licenses/LICENSE-2.0). All code and any additional files referenced in the manuscript are available at the GitHub repository https://github.com/superphy/version-1.

## Abbreviations

AMR: anti-microbial resistance
CARD: comprehensive antibiotic resistance database
DNA: deoxyribonucleic acid
GMOD: generic model organism database
SNP: single-nucleotide polymorphism
Stx: Shiga-toxin
WGS: whole-genome sequencing
